# Transmission Patterns of Seasonal Influenza in China between 2010 and 2018

**DOI:** 10.3390/v14092063

**Published:** 2022-09-17

**Authors:** Hao Lei, Lei Yang, Gang Wang, Chi Zhang, Yuting Xin, Qianru Sun, Bing Zhang, Tao Chen, Jing Yang, Weijuan Huang, Modi Xu, Yu Xie, Yinghan Wang, Pei Xu, Litao Sun, Deyin Guo, Xiangjun Du, Dayan Wang, Yuelong Shu

**Affiliations:** 1 School of Public Health (Shenzhen), Sun Yat-sen University, Guangzhou 510275, China; 2 School of Public Health (Shenzhen), Shenzhen Campus of Sun Yat-sen University, Shenzhen 518107, China; 3 School of Public Health, Zhejiang University, Hangzhou 310058, China; 4 Key Laboratory for Medical Virology, National Health Commission, National Institute for Viral Disease Control and Prevention, Collaboration Innovation Center for Diagnosis and Treatment of Infectious Diseases, Chinese Center for Disease Control and Prevention, Beijing 102206, China; 5 Guangzhou First People’s Hospital, School of Medicine, South China University of Technology, Guangzhou 510180, China; 6 The Second Affiliated Hospital of Kunming Medical University, Kunming 650000, China; 7 School of Medicine, Shenzhen Campus of Sun Yat-sen University, Shenzhen 518107, China; 8 School of Medicine, Sun Yat-sen University, Guangzhou 510275, China; 9 Key Laboratory of Tropical Disease Control, (Sun Yat-sen University) Ministry of Education, Guangzhou 510030, China; 10 Institute of Pathogen Biology of Chinese Academy of Medical Science (CAMS)/Peking Union Medical College (PUMC), Beijing 100730, China

**Keywords:** seasonal influenza, transmission, initiating area, driver, China

## Abstract

Background Understanding the transmission source, pattern, and mechanism of infectious diseases is essential for targeted prevention and control. Though it has been studied for many years, the detailed transmission patterns and drivers for the seasonal influenza epidemics in China remain elusive. Methods In this study, utilizing a suite of epidemiological and genetic approaches, we analyzed the updated province-level weekly influenza surveillance, sequence, climate, and demographic data between 1 April 2010 and 31 March 2018 from continental China, to characterize detailed transmission patterns and explore the potential initiating region and drivers of the seasonal influenza epidemics in China. Results An annual cycle for influenza A(H1N1)pdm09 and B and a semi-annual cycle for influenza A(H3N2) were confirmed. Overall, the seasonal influenza A(H3N2) virus caused more infection in China and dominated the summer season in the south. The summer season epidemics in southern China were likely initiated in the “Lingnan” region, which includes the three most southern provinces of Hainan, Guangxi, and Guangdong. Additionally, the regions in the south play more important seeding roles in maintaining the circulation of seasonal influenza in China. Though intense human mobility plays a role in the province-level transmission of influenza epidemics on a temporal scale, climate factors drive the spread of influenza epidemics on both the spatial and temporal scales. Conclusion The surveillance of seasonal influenza in the south, especially the “Lingnan” region in the summer, should be strengthened. More broadly, both the socioeconomic and climate factors contribute to the transmission of seasonal influenza in China. The patterns and mechanisms revealed in this study shed light on the precise forecasting, prevention, and control of seasonal influenza in China and worldwide.

## 1. Introduction

As a major cause of human morbidity and mortality every year, seasonal influenza epidemics lead to about 88,100 excess respiratory deaths annually in China [1]. Seasonal influenza epidemics are mostly initiated from a source region and then spread worldwide rather than descending from a previous epidemic in the same region [2]. On a global scale, influenza A viruses circulating in Southeast Asia have been proposed as the source of seasonal epidemics in other regions [2,3]. In China, new antigenic variants usually first emerge from the south [4]. China is a geographically, economically, and climatologically diverse country, and prior studies have suggested that northern China experienced influenza epidemics only in the winter–spring months, while in southern China, influenza is prevalent throughout the year with a clear peak in both the summer and winter [5,6]. However, a detailed transmission pattern and its potential mechanism are still missing, which are critical for designing targeted surveillance and control strategies.

Prior studies have suggested that influenza spatial spread results from the combined effect of climate factors and population mobility patterns [6,7,8]. Experimental studies suggest that influenza viruses favor low humidity and low temperature [9]. A human population-level epidemiological study also indicated that absolute humidity drove the seasonal variations of influenza transmission [10] and the 2009 pandemic influenza spatial transmission [11] in the continental United States. The roles of human mobility in the spatial spread of seasonal influenza have been explored by several studies. On a global scale, the worldwide air transportation network serves as the predominant channel for the dissemination of the pandemic and seasonal influenza viruses [2,12,13]. On the regional scale, short-distance work commutes are a major driver of the spread of seasonal outbreaks in the US, though longer-range air traffic has been implicated in playing an important role as well [14,15,16,17]. Different from the transportation pattern in the United States, where personal vehicular movement and long-range airline travel play the predominant roles in the within-region and inter-regional transport, respectively [16], in China, passenger transport, particularly the high-speed railway, has developed rapidly in the past decade and could play an important role in the inter-province human mobility.

In this study, based on the new comprehensive surveillance data of seasonal influenza in China between 2010 and 2018, the more detailed transmission patterns of seasonal influenza in continental China were explored, in addition to the potential mechanisms based on a combination of epidemiological, genetic, socioeconomic, and meteorological data. 

## 2. Methods

### 2.1. Influenza Surveillance Dataset 

The national sentinel hospital-based influenza-like illness (ILI) surveillance system in China was established in 2000, with 99 sentinel surveillance hospitals by 2008 [18]. After the 2009 influenza pandemic, the system was expanded to 554 sentinel hospitals with all 31 provinces implementing year-round surveillance. The weekly reports of influenza cases were downloaded from the Chinese Centre for Disease Control and Prevention (http://www.chinaivdc.cn/cnic/, accessed on 24 October 2019). The dataset is based on the number of specimens tested in 554 sentinel hospitals located in 31 Chinese administrative regions; here, the major administrative regions included provinces, autonomous regions, and municipalities, and for simplicity, termed “province” hereafter. Tibet is excluded from our analysis because of its relatively poor surveillance system. The period from 1 April 2010 to 31 March 2018 was studied in this work, as the quality of the surveillance data improved dramatically after the 2009 influenza A(H1N1) pandemic [19]. The dataset provided the weekly number of visits in each hospital, the number of influenza-like illness (ILI) cases, the number of specimens tested, the number of laboratory-confirmed influenza cases for seasonal influenza A(H1N1)pdm09, A(H3N2), B/Yamagata, and B/Victoria (Table 1). According to the *Chinese National Influenza Surveillance Guideline*, an ILI case was defined as a patient with a fever of ≥38 °C, accompanied by cough or sore throat [20]. 

### 2.2. Sequence Data

The hemagglutinin (HA) nucleotide sequences of the seasonal A(H3N2) influenza viruses from diverse regions of Mainland China between 1 April 2010 and 31 March 2018 were downloaded from the Global Initiative on Sharing Avian Influenza Data (GISAID, https://www.gisaid.org, accessed on 24 October 2019). The sequences were then aligned with MUSCLE v3.7 using its default settings. Only those sequences with the full date (year, month, and day) and location (province) information were used. Furthermore, the outliers and redundant data were manually removed. Finally, 1403 HA sequences of H3N2 were analyzed in this study. The sequence datasets of the A(H1N1)pdm09, B/Yamagata, and B/Victoria influenza viruses were obtained and processed in the same way, with sequence numbers 828, 647, and 431, respectively.

### 2.3. Climate and Demographic Data

To assess the role of the putative drivers of spatial influenza transmission, the province-level demographic and climate were collected during the study period. For the demographic data, the railway data as a proxy of inter-provincial human mobility were used [21,22,23] (see Supplementary Materials Part S1 for more details). For the climate data, temperature and relative humidity were used. The daily climate data for each participating weather station during the study period were obtained from the website of the China Meteorological Administration (http://data.cma.cn, accessed on 24 October 2019). The province-level daily meteorological indicators were calculated as the averages of the data in all the weather stations in that province, and the weekly indicators were calculated as averages of the daily values in that week.

### 2.4. Time Series Analysis

From the data streams, same as previous studies, a proxy for the weekly strain-specific incidence rate (henceforth simply termed “incidence rate”) of each subtype was used, a measurement more precisely representing influenza infections [24,25,26]. The weekly incidence rate for each subtype was defined as the ILI rate among the visiting patients in sentinel hospitals multiplied by the viral detection rate for each subtype individually. When the number of specimens is small, the incidence rate for a specific week could be biased as an outlier. To remove the outliers, a threshold of 30 was used for the number of specimens, i.e., when the weekly number of specimens was lower than 30, the value of the outlier was replaced by the mean value of the same week in other years. 

Since China is located in the Northern Hemisphere, an epidemiological annual cycle was defined as the period from 1 April (calendar week 14, epidemiological week 1 in our notation) to 31 March of the next year [6]. Several studies have reported that seasonal influenza epidemics experience a semi-annual cycle pattern in southern China and an annual cycle in northern China [5,6], so the annual cycle was also divided into the summer season and winter season, which are from calendar week 14 to 39 and from calendar week 40 to week 13 of the next year, respectively [6]. For simplicity, an epidemic was referred to as a summer wave or a winter wave if the peak time occurred in the summer season or winter season defined above. The traditional wavelet analysis was also applied to explore the seasonality of seasonal influenza in China (see Supplementary Materials Part S1 for more details). A cumulative incidence rate was defined as the sum of the weekly incidence rate for a specific time period [24], and the prevalence of a specific subtype at a given period was defined as the proportion of the cumulative incidence rate of this subtype during this period.

The onset of seasonal influenza for a specific region was defined as the first of three consecutive weeks with increasing smoothed weekly incidence rates and exceeding a prescribed baseline [11,26]. Since fluctuations in the surveillance data may play a particular role in estimating the onset timing of an epidemic, a 3-week moving average was used to smooth the surveillance data (see Supplementary Materials Part S1 for more details). The baseline was set as the 40% quantile of the non-zero weekly incidence rate of an influenza strain or a combined strain [26]. The ending of an epidemic was defined as the first of three consecutive weeks with a smoothed weekly incidence rate below the baseline.

### 2.5. Evolutionary Analysis

To characterize the transmission pattern between southern and northern China in genomic ways, the most parsimonious evolutionary path analysis was used [27], which defines each virus’s most likely ancestor to have the highest sequence similarity among all older viruses. All the most parsimonious evolutionary paths were traced, and the transition events between northern and southern China both within the same season or across the different seasons were recorded. In order to avoid sampling bias, bootstrap was applied 100 times via random sampling to 3 sequences per season from each province each time. The Hamming distance was used as an indicator of the genetic distance in this study.

### 2.6. Clustering Analysis

Hierarchical clustering with Ward’s minimum variance method [28] was used to identify regional clusters, relying on the squared Euclidian pairwise difference (epidemiological distance) between the Spearman correlations of incidence rate time series [29] among the provinces as the distance metrics [30].

### 2.7. Effective Distance

The identification of the initiating area of an epidemic from an epidemiological approach relied on the concept that an epidemic’s arrival time in one region is linearly related to the effective distance from the source [12,31]. An effective distance between region i and region j $d_{ij}$ is defined as

$$d_{ij}=1-logT_{ij}$$

where $T_{ij}=T_{ij}/T_{j}$, $T_{ij}$ is the number of those individuals who traveled from region i to region j per time unit, and $T_{j}=\sum_{i=1}^{N}T_{ij}$. Additionally, in this study, the number of people traveling by railway was used to estimate inter-province transport in China (see Supplementary Materials Part S1 for more details).

### 2.8. Statistical and Regression Analysis

To reveal the determinants for the spatial spread of influenza, the relative contributions of different potential drivers were quantified using a fixed-effect linear regression model, with an interaction term between temperature and relative humidity, which were believed to be correlated. A set of independent variables were firstly normalized via min–max scaling. From both the spatial scale and temporal scale, the dependent variable was the onset time, and it could be compared with the onset times of the region of origin or the first epidemiological year as references. The independent variables were the daily human mobility from the potential regional origin, the differences in the mean temperature and humidity in the summer months, and the latitude and longitude. The epidemiological year 2010 was excluded for analysis because the epidemics in the summer months this year were much later than those in other years, partly due to the influence of the 2009 influenza A(H1N1) pandemic. Their goodness-of-fit was measured with the Akaike information criterion (AIC), and a backward-reduced AIC-variable selection procedure tested whether the best fitting bivariate relationship could be further improved by removing the independent variables. The statistical difference was tested using *t*-tests, and the two-sided *p* values are reported. The correlation coefficient was determined based on the Pearson correlation method. The data were analyzed using R software, version 3.6.1.

## 3. Results

### 3.1. Surveillance of Seasonal Influenza in China between 2010 and 2018

During the study period from 1 April 2010 to 31 March 2018, the weekly average number of the specimens tested by province was 258 (compared with 42 between 2005 and 2011; data from a previous report), with the most intense sampling in Guangdong Province (563 on average) and thinnest sampling in Hainan Province (109 on average). This level of sampling corresponded to 3.07 respiratory samples tested on average per year per 10,000 populations in China (compared with 1.81 between 2005 and 2011). On average, 39 influenza virus-positive specimens were identified per week by province (compared with 7 between 2005 and 2011) (Table 1). 

### 3.2. Epidemiology of Seasonal Influenza in China

**Figure 1:**
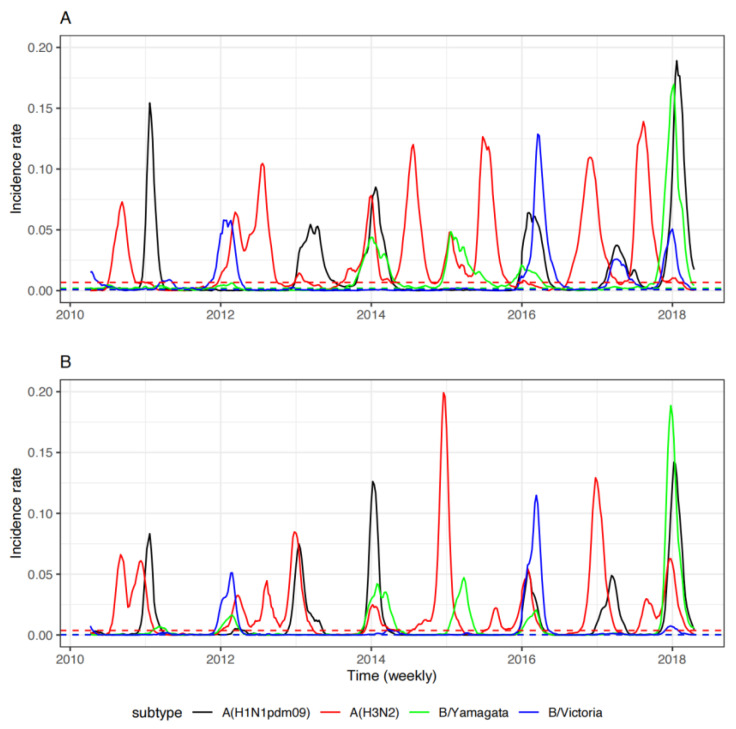
Weekly incidence rate for four seasonal influenza strains in southern China (**A**) and northern China (**B**). Horizontal dash line is the baseline for each strain (see Methods for more details).

Based on the incidence rate data from northern and southern China, in the 8 years spanning from 1 April 2010 to 31 March 2018 (Figure 1), six epidemic waves of influenza A(H1N1)pdm09 were identified in both southern and northern China, all in the winter season. In the years 2012 and 2015, there was no influenza A(H1N1)pdm09 epidemic wave in the winter season. For influenza A(H3N2), there were five summer and four winter epidemic waves in southern China, and northern China experienced three summer and seven winter influenza A(H3N2) epidemic waves. There were four and five epidemic waves of influenza B/Yamagata in southern and northern China, respectively, all in the winter season. There were five and three winter epidemic waves of influenza B/Victoria in southern China and northern China, respectively. Our detailed wavelet analysis confirmed that the influenza A(H1N1)pdm09 and B viruses followed a single annual cycle with peaks during the winter season for both northern and southern China, while the influenza A(H3N2) virus experienced a semi-annual cycle in southern China, with additional peaks in the summer season (Supplementary Figure S1). 

**Figure 2:**
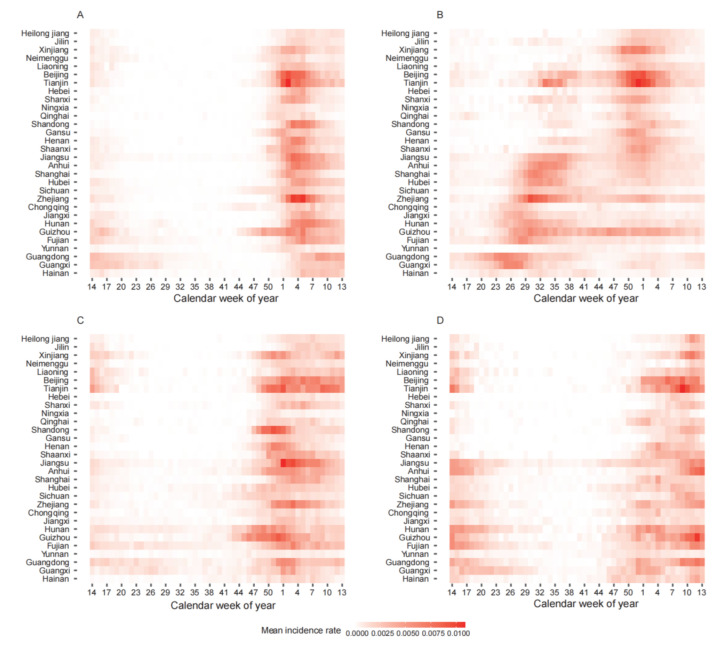
Heatmap for the mean weekly incidence rate of influenza (**A**) A(H1N1)pdm09, (**B**) A(H3N2), (**C**) B/Yamagata, and (**D**) B/Victoria for provinces in China, sorted by increasing latitude from bottom to top.

The heatmaps for the mean weekly incidence rate of influenza A(H1N1) pdm09, A(H3N2), B/Yamagata, and B/Victoria at the province level showed the same consistent pattern: influenza A(H1N1)pdm09 and B experienced epidemics in winter, and for the influenza A(H3N2) virus, there was an additional summer peak in the south (Figure 2). During the study period, the disease burden caused by the influenza A(H3N2) virus was the largest (the proportions of A(H1N1)pdm09, A(H3N2), B/Yamagata, and B/Victoria were 27.1%, 43.6%, 17.4%, and 11.9%, respectively). In the summer epidemics, for the 15 provinces in southern China, on average, the influenza A(H3N2) virus contributed to 69.1% of the epidemics (ranging from 43.2% to 87.3%) (Supplementary Figure S2A). For the winter epidemics, on average, influenza A(H1N1)pdm09, A(H3N2), and B each contributed to one-third, respectively (Supplementary Figure S2B). At the same time, for the winter epidemics, the prevalence of influenza B decreased toward the north (R^2^ = 0.53, *p* =4.9 × 10^−6^), while the prevalence of influenza A(H3N2) increased with latitude (R^2^ = 0.74, *p* = 1.3 × 10^−9^).

### 3.3. Spatial Transmission of Seasonal Influenza in China

Based on the epidemiological surveillance data, there was a latitude gradient for the onset timing of influenza A(H3N2) virus epidemics in southern China in the summer months, with regions in the lower latitude starting the epidemic earlier (R^2^ = 0.50, *p* = 0.0032, Table 2). However, there was no longitude gradient (R^2^ = 0.05, *p* = 0.43, Table 2). For the winter–spring waves, there was a weak longitude gradient in the influenza A(H3N2) onset timing (R^2^ = 0.13, *p* = 0.047) and a latitude gradient in the influenza B/Yamagata onset timing (R^2^ = 0.51, *p* = 8.7 × 10^−6^, Table 2). 

Based on the evolutionary relationship among viruses, for influenza A(H1N1)pdm09, B/Yamagata, and B/Victoria, which mainly experienced epidemics in the winter months, the regions in the south played more important seeding roles based on the fact that more transition events were observed from the south to the north (Supplementary Tables S1–S3). For influenza A(H3N2) in the winter from both the north and south, the viruses mostly originated from the previous summer season in southern China, rather than from the previous winter season (Supplementary Table S4). Similarly, the regions in the south play more important roles in maintaining the circulation of the influenza A(H3N2) virus during both the summer and winter seasons (Supplementary Table S5). 

### 3.4. Initiating Area and Drivers for Transmission of Influenza A(H3N2) in the Summer

**Figure 3:**
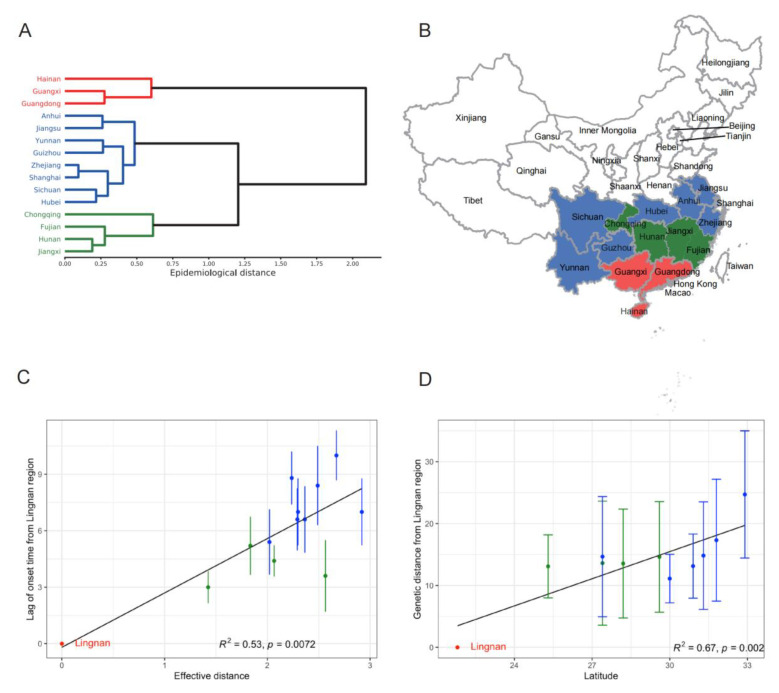
Initiating area of seasonal influenza A(H3N2) in southern China during summer season: (**A**) hierarchical clustering for provinces based on epidemiological distance; (**B**) geographical map of the three epidemiological regions identified in (**A**); (**C**) effective distance against lag of onset time from “Lingnan” region for influenza A(H3N2) epidemic; (**D**) mean genetic distance from the initiating “Lingnan” region for different provinces in southern China. Dark line is the linear fit line, and error bar represents the standard deviation.

The three most southern provinces (Guangdong, Guangxi, and Hainan, defined as the “Lingnan” region) shared similar influenza epidemiological patterns and were clustered together (Figure 3A,B). Based on the spatial gradient observed above for the influenza A(H3N2) onset time during the summer season in the south, the “Lingnan” region was the potential initiating area of influenza A(H3N2) epidemics. We tested our hypothesis based on two analyses: One is based on the concept of effective distance, and the other one is based on the viruses’ evolutionary relationship (see Section 2 for more details). There was a significant positive relationship between the influenza A(H3N2) onset time and the effective distance from the “Lingnan” region (Figure 3C). Additionally, the genetic distance from the “Lingnan” region was linearly increased with the latitude (Figure 3D), both of which supported our hypothesis.

According to the regression analysis between the onset time and the three candidate drivers, the climate factors became the statistically significant driver for the spread of seasonal influenza A(H3N2) virus on the spatial scale (Table 3, *p* < 0.05). The positive correlation means that the larger the difference in the mean temperature and relative humidity in the originating “Lingnan” region, the later the arrival of the epidemics. Additionally, from a temporal scale, human mobility became a significant driver in addition to the relative humidity (Table 3, *p* < 0.05). The negative correlation regarding human mobility indicated that the greater the degree of human mobility from a region of origin, the earlier the arrival of the influenza epidemics. Moreover, only relative humidity was the statistically significant driver in both the spatial and temporal scales.

## 4. Discussion

The motivation for this study was to reveal the detailed transmission patterns of seasonal influenza to assist the ongoing efforts for real-time forecasting and targeted control strategies in China. Based on the availability of rich surveillance data between 2010 and 2018, a comprehensive analysis of the seasonal influenza epidemics in China was carried out, and the pattern and mechanisms were identified. There was a single annual peak for influenza A(H1N1)pdm09 and B in the winter season and a semi-annual periodicity for A(H3N2) in southern China with an additional peak in the summer. The “Lingnan” region in the southern continental area of China, which includes Hainan, Guangxi, and Guangdong Provinces, was the potential initiating region for seasonal influenza A(H3N2). Every year, seasonal influenza A(H3N2) epidemics started around April in the “Lingnan” region and spread to other provinces in southern China within 2–3 months, driven by both the climate and human mobility. When a suitable climate arrived, the influenza A(H3N2) viruses circulated in the summer season from southern China were rapidly transmitted to the whole country, leading to the winter epidemics. Overall, the regions in the south played more important roles in maintaining the circulation of all the seasonal influenza strains in China, which is well-aligned with the source–sink models proposed previously [3,32,33]. Thus, influenza surveillance in the “Lingnan” region should be strengthened. Additionally, the surveillance data from the “Lingnan” region could be used to inform the arrival time and intensity of influenza epidemics in other regions in southern China a few weeks ahead, combined with the human mobility and climate data. The influenza surveillance information in the “Lingnan” region could also be used to predict the dominant influenza subtype in other provinces for the same epidemiological year, which could provide a basis for better recommendations for influenza vaccine strains and the use of antiviral drugs. These are helpful for the precise prediction and prevention of seasonal influenza in China.

Prior work has suggested that influenza viruses favor low humidity and low temperature, as indicated in an experimental study [9] as well as a human population-level epidemiological study [34]. Based on the data from our study, there was another suitable climate parameter in the summer for the effective transmission of H3N2, with high temperatures (20 °C–30 °C) and high levels of relative humidity (75–90%) (Supplementary Figure S3A). Furthermore, there was a clear spatial gradient regarding the spread of the seasonal influenza A(H3N2) virus initiated by the “Lingnan” region, which was mostly determined by the climate factors, i.e., the regions meeting favorable climate conditions were affected first (Supplementary Figure S3B). The seasonal influenza epidemics in the United States also likely originated in the South, including cities such as Miami and Houston [15]. Brazil also experiences a transmission gradient from the tropical to temporal regions [7]. These patterns are consistent with the previous knowledge of the importance of tropical regions for initiating seasonal influenza epidemics [2,3].

The role of human mobility in the spatial spread of seasonal influenza is crucial. In this study, we showed that the increasing volume of human travel has the potential to accelerate influenza spread on a temporal scale in China (Table 3). Additionally, northern China does not have favorable climate conditions (Supplementary Figure S3A) for the seasonal influenza A(H3N2) virus in the summer, and the summer peaks in northern China were always followed by the intense summer peaks of influenza A(H3N2) in southern China (Figure 1), implying that the summer peaks in northern China might be caused by the population from southern China.

In this study, we only analyzed the influenza surveillance data between 2010 and 2018, and the emergency of the COVID-19 pandemic in 2020 largely disturbed the influenza transmission due to the non-pharmaceutical interventions (NPIs) used to control the COVID-19 pandemic [35,36]. However, we believe that the results from this study could also be applicable after the COVID-19 pandemic when the non-pharmaceutical interventions are lifted. Unfortunately, we could not perform a recent analysis from the start of the pandemic due to the lack of data, as influenza epidemics almost disappeared in China during this period.

Our study is prone to several limitations. The mobility data we used were based on inter-province railway transportation data. We argue that although road travel plays an important role in population movement, it is mainly short-distanced and is not increasing. Although human mobility due to air travel has increased, it is still several magnitudes less than rail travel. Future work needs to collect more detailed population movement data and comprehensively peruse all those data. Additionally, socioeconomic events including school opening and population size and density need to be considered in future analyses to reveal more detailed transmission patterns and additional potential drivers [11,15,17]. Additionally, the potential interaction between the different strains of seasonal influenza was reported [24] and observed by the data (i.e., a reverse spatial gradient between influenza B and A(H3N2) in the winter), and these confounding factors should be considered in the future. Finally, evolutionary evidence is supplementary to epidemiological support, and more genetic sequences are needed for robust and detailed analyses.

## Figures and Tables

**Table 1:** Background characteristics of the 30 provinces involved in influenza surveillance and information on influenza sampling intensity, 2010–2018, China.

Province ^a^	Cities (Hospitals) ^b^	Population Size ^c^ (M)	Latitude	Longitude	Mean Weekly Temperature in Summer (°C)	Mean Weekly Relative Humidity in Summer (%)	Mean Weekly Temperature in Winter (°C)	Mean Weekly Relative Humidity in Winter (%)	Mean Weekly Specimens Tested	Mean Weekly Influenza Positive
Hainan	5 (6)	4.89	19.6	110.1	27.8	81.7	22.0	82.6	109	12
Guangxi	14 (17)	48.12	22.9	108.4	26.5	79.1	16.6	76.2	318	52
Guangdong	20 (27)	89.20	22.9	113.4	26.7	81.1	17.5	75.0	563	90
Yunnan	14 (17)	44.57	24.8	103.0	21.1	73.7	13.5	68.5	305	29
Fujian	9 (15)	39.11	25.3	118.8	24.7	79.7	14.1	76.9	321	56
Guizhou	8 (13)	32.27	27.4	106.8	21.7	78.5	10.3	79.6	220	32
Hunan	14 (23)	45.55	27.4	113.0	24.2	77.23	10.9	76.6	375	46
Jiangxi	11 (15)	46.22	28.2	115.3	24.9	78.7	11.8	77.4	242	40
Chongqing	1 (7)	33.90	29.6	106.6	24.1	75.7	11.7	78.9	113	22
Zhejiang	12 (16)	50.37	30.0	120.4	24.2	77.8	11.3	74.1	302	61
Sichuang	21 (31)	83.21	30.2	104.0	18.0	70.5	7.0	62.9	347	50
Hubei	13 (18)	57.17	30.9	112.6	23.7	76.9	9.7	73.7	314	50
Shanghai	1 (19)	24.18	31.3	121.5	24.2	72.2	10.5	69.5	317	82
Anhui	17 (25)	64.16	31.8	117.5	23.4	76. 7	8.6	71.8	371	61
Jiangsu	13 (29)	80.29	32.9	118.6	23.3	75.7	8.2	70.4	543	73
Shaanxi	10 (18)	39.76	34.3	112.8	20.1	68.1	4.4	62.6	211	32
Henan	18 (22)	95.59	34.7	113.1	23.2	69.1	7.3	61.7	237	39
Gansu	14 (19)	23.26	35.6	104.7	17.0	53.5	−0.3	52.1	204	31
Shandong	17 (27)	100.05	36.3	118.4	21.6	70.3	5.1	61.2	318	46
Qinghai	9 (14)	5.87	36.6	101.8	10.2	55.0	−4.7	42.6	126	13
Ningxia	5 (9)	6.82	37.6	106.0	17.8	54.2	0.1	52.0	115	14
Shanxi	11 (17)	37.02	37.8	112.8	19.2	59.9	1.2	53.5	183	31
Hebei	10 (24)	70.73	38.1	115.8	20.7	62.1	1.2	54.6	258	37
Tianjin	1 (10)	15.57	39.2	117.2	22.6	62.4	3.5	54.4	109	22
Beijing	1 (11)	21.71	39.9	116.4	21.5	59.7	1.8	49.9	215	37
Liaoning	14 (21)	41.97	40.7	122.6	19.3	68.4	−1.4	58.5	255	23
Neimenggu	12 (19)	25.28	40.8	110.8	16.8	49.1	−7.2	53.1	161	19
Xinjiang	13 (16)	20.57	43.8	87.6	19.4	43.1	−2.2	59.1	208	26
Jilin	9 (13)	26.16	44.1	125.4	17.2	67.4	−6.4	62.9	147	18
Heilongjiang	13 (20)	35.85	46.1	126.2	16.0	68.8	−10.7	66.0	225	24

^a^ Sorted by increasing latitude from top to bottom. ^b^ Number of cities and hospitals participating in surveillance. ^c^ Number of people in participating cities.

**Table 2:** Latitude and longitude gradient analysis for onset time of influenza A(H1N1) pdm09, A(H3N2), B/Yamagata, and B/Victoria in summer and winter seasons, respectively.

	R^2^ (*p* Value)			
	Summer Season		Winter Season	
	Latitude	Longitude	Latitude	Longitude
A(H3N2)	0.50 (3.2 × 10^−3^)	0.05 (4.3 × 10^−1^)	0.11 (7.3 × 10^−2^)	0.13 (4.7 × 10^−2^)
A(H1N1)pdm09	NA	NA	0.02 (5.1 × 10^−1^)	0.05 (2.6 × 10^−1^)
B/Yamagata	NA	NA	0.51 (8.7 × 10^−6^)	0.086 (1.2 × 10^−1^)
B/Victoria	NA	NA	0.09 (1.1 × 10^−1^)	0.01 (5.9 × 10^−1^)

**Table 3:** Regression analysis between onset time and temperature, relative humidity, and transportation of seasonal influenza A(H3N2) epidemics on spatial and temporal scales.

Predictors	Spatial Scale		Temporal Scale	
	Coefficients (Standard Error)	*p* Value	Coefficients (Standard Error)	*p* Value
Normalized temperature	20.1 (3.3)	2.0 × 10^−7^	7.2 (5.4)	1.8 × 10^−1^
Normalized relative humidity	13.6 (2.9)	2.2 × 10^−5^	10.1 (4.7)	3.8 × 10^−2^
Normalized transport volume	−0.4 (1.9)	8.2 × 10^−1^	−7.4 (3.0)	1.9 × 10^−2^
Interaction term	−0.5 (1.6)	7.5 × 10^−1^	−14.5 (7.9)	7.5 × 10^−2^

## Data Availability

Due to the potentially sensitive information included, the original dataset is not public and is available from the corresponding author upon reasonable request. The descriptions of the model are available in the Supplementary Materials.

## Supplementary Materials

The following supporting information can be downloaded at: https://www.mdpi.com/article/10.3390/v14092063/s1, Part S1: Methods; Table S1: Transmission analysis within the same winter season for influenza A(H1N1)pdm09 based on sequences; Table S2: Transmission analysis within the same winter season for influenza B/Yamagata based on sequences; Table S3: Transmission analysis within the same winter season for influenza B/Victoria based on sequences; Table S4: Transmission analysis between seasons for influenza A(H3N2) based on sequences; Table S5: Transmission analysis within the same season for influenza A(H3N2) based on sequences; Figure S1: Wavelet analysis of the seasonality of influenza (A) A(H1N1)pdm09, (B) A(H3N2), (C) B/Yamagata, and (D)B/Victoria in southern (left) and northern (right) China, respectively; Figure S2: Prevalence of influenza strains in China: (A) summer season in southern China and (B) winter season across China. Provinces were sorted by increasing latitude from bottom to top; Figure S3: Environmental space for seasonal influenza A(H3N2) in China: (A) environment space levels illustrated based on mean temperature and relative humidity. The mean values in the summer season for provinces in northern China were marked as a black dot; (B) density curve for the three regions distinguished by different colors in Figure 3A; Figure S4: Number of passengers traveled by railway, road, water, and air from 2010 to 2017.

## References

[1] Li L., Liu Y., Wu P., Peng Z., Wang X., Chen T., Wong J.Y.T., Yang J., Bond H.S., Wang L. (2019). Influenza-Associated Excess Respiratory Mortality in China, 2010-15: A Population-Based Study. Lancet Public Health.

[2] Russell C.A., Jones T.C., Barr I.G., Cox N.J., Garten R.J., Gregory V., Gust I.D., Hampson A.W., Hay A.J., Hurt A.C. (2008). The Global Circulation of Seasonal Influenza A (H3N2) Viruses. Science.

[3] Rambaut A., Pybus O.G., Nelson M.I., Viboud C., Taubenberger J.K., Holmes E.C. (2008). The Genomic and Epidemiological Dynamics of Human Influenza A Virus. Nature.

[4] Du X., Dong L., Lan Y., Peng Y., Wu A., Zhang Y., Huang W., Wang D., Wang M., Guo Y. (2012). Mapping of H3N2 Influenza Antigenic Evolution in China Reveals a Strategy for Vaccine Strain Recommendation. Nat. Commun..

[5] Shu Y.-L., Fang L.-Q., de Vlas S.J., Gao Y., Richardus J.H., Cao W.-C. (2010). Dual Seasonal Patterns for Influenza, China. Emerg. Infect Dis..

[6] Yu H., Alonso W.J., Feng L., Tan Y., Shu Y., Yang W., Viboud C. (2013). Characterization of Regional Influenza Seasonality Patterns in China and Implications for Vaccination Strategies: Spatio-Temporal Modeling of Surveillance Data. PLoS Med.

[7] Alonso W.J., Viboud C., Simonsen L., Hirano E.W., Daufenbach L.Z., Miller M.A. (2007). Seasonality of Influenza in Brazil: A Traveling Wave from the Amazon to the Subtropics. Am. J. Epidemiol.

[8] Tamerius J.D., Shaman J., Alonso W.J., Alonso W.J., Bloom-Feshbach K., Uejio C.K., Comrie A., Viboud C. (2013). Environmental Predictors of Seasonal Influenza Epidemics across Temperate and Tropical Climates. PLoS Pathog..

[9] Lowen A.C., Mubareka S., Steel J., Palese P. (2007). Influenza Virus Transmission Is Dependent on Relative Humidity and Temperature. PLoS Pathog..

[10] Shaman J., Pitzer V.E., Viboud C., Grenfell B.T., Lipsitch M. (2010). Absolute Humidity and the Seasonal Onset of Influenza in the Continental United States. PLoS Biol..

[11] Gog J.R., Ballesteros S., Viboud C., Simonsen L., Bjornstad O.N., Shaman J., Chao D.L., Khan F., Grenfell B.T. (2014). Spatial Transmission of 2009 Pandemic Influenza in the US. PLoS Comput. Biol..

[12] Brockmann D., Helbing D. (2013). The Hidden Geometry of Complex, Network-Driven Contagion Phenomena. Science.

[13] Khan K., Arino J., Hu W., Raposo P., Sears J., Calderon F., Heidebrecht C., Macdonald M., Liauw J., Chan A. (2009). Spread of a Novel Influenza A (H1N1) Virus via Global Airline Transportation. N. Engl. J. Med..

[14] Pei S., Kandula S., Yang W., Shaman J. (2018). Forecasting the Spatial Transmission of Influenza in the United States. Proc. Natl. Acad. Sci. USA.

[15] Charu V., Zeger S., Gog J., Bjørnstad O.N., Kissler S., Simonsen L., Grenfell B.T., Viboud C. (2017). Human Mobility and the Spatial Transmission of Influenza in the United States. PLoS Comput. Biol..

[16] Brownstein J.S., Wolfe C.J., Mandl K.D. (2006). Empirical Evidence for the Effect of Airline Travel on Inter-Regional Influenza Spread in the United States. PLoS Med..

[17] Viboud C., Bjørnstad O.N., Smith D.L., Simonsen L., Miller M.A., Grenfell B.T. (2006). Synchrony, Waves, and Spatial Hierarchies in the Spread of Influenza. Science.

[18] Feng L., Shay D.K., Jiang Y., Zhou H., Chen X., Zheng Y., Jiang L., Zhang Q., Lin H., Wang S. (2012). Influenza-Associated Mortality in Temperate and Subtropical Chinese Cities, 2003–2008. Bull World Health Organ.

[19] Liu X.-X., Li Y., Zhu Y., Zhang J., Li X., Zhang J., Zhao K., Hu M., Qin G., Wang X.-L. (2017). Seasonal Pattern of Influenza Activity in a Subtropical City, China, 2010-2015. Sci. Rep..

[20] Shu Y., Song Y., Wang D., Greene C.M., Moen A., Lee C.K., Chen Y., Xu X., McFarland J., Xin L. (2019). A Ten-Year China-US Laboratory Collaboration: Improving Response to Influenza Threats in China and the World, 2004-2014. BMC Public Health.

[21] U.S. Influenza Surveillance: Purpose and Methods | CDC. https://www.cdc.gov/flu/weekly/overview.htm.

[22] (2018). China Statistical Yearbook. http://www.stats.gov.cn/tjsj/ndsj/2018/indexeh.htm..

[23] (2016). China Transport. Statistical Yearbook 2016.

[24] Goldstein E., Cobey S., Takahashi S., Miller J.C., Lipsitch M. (2011). Predicting the Epidemic Sizes of Influenza A/H1N1, A/H3N2, and B: A Statistical Method. PLoS Med..

[25] Shaman J., Karspeck A., Yang W., Tamerius J., Lipsitch M. (2013). Real-Time Influenza Forecasts during the 2012-2013 Season. Nat. Commun..

[26] Yang W., Cowling B.J., Lau E.H.Y., Shaman J. (2015). Forecasting Influenza Epidemics in Hong Kong. PLoS Comput. Biol..

[27] Chan J., Holmes A., Rabadan R. (2010). Network Analysis of Global Influenza Spread. PLoS Comput. Biol..

[28] Ward J.H. (1963). Hierarchical Grouping to Optimize an Objective Function. Null.

[29] Lu F.S., Hattab M.W., Clemente C.L., Biggerstaff M., Santillana M. (2019). Improved State-Level Influenza Nowcasting in the United States Leveraging Internet-Based Data and Network Approaches. Nat. Commun..

[30] Szekely G.J., Rizzo M.L. (2005). Hierarchical Clustering via Joint Between-Within Distances: Extending Ward’s Minimum Variance Method. J. Classif..

[31] Smith D.J., Lapedes A.S., de Jong J.C., Bestebroer T.M., Rimmelzwaan G.F., Osterhaus A.D.M.E., Fouchier R.A.M. (2004). Mapping the Antigenic and Genetic Evolution of Influenza Virus. Science.

[32] Wen F., Bedford T., Cobey S. (2016). Explaining the Geographical Origins of Seasonal Influenza A (H3N2). Proc. Biol. Sci..

[33] Aris-Brosou S. (2014). Inferring Influenza Global Transmission Networks without Complete Phylogenetic Information. Evol. Appl..

[34] Shaman J., Kohn M. (2009). Absolute Humidity Modulates Influenza Survival, Transmission, and Seasonality. Proc. Natl. Acad. Sci. USA.

[35] Qiu Z., Cao Z., Zou M., Tang K., Zhang C., Tang J., Zeng J., Wang Y., Sun Q., Wang D. (2022). The Effectiveness of Governmental Nonpharmaceutical Interventions against COVID-19 at Controlling Seasonal Influenza Transmission: An Ecological Study. BMC Infect. Dis..

[36] Lei H., Xu M., Wang X., Xie Y., Du X., Chen T., Yang L., Wang D., Shu Y. (2020). Nonpharmaceutical Interventions Used to Control COVID-19 Reduced Seasonal Influenza Transmission in China. J. Infect Dis..

