# Supplementary material for: Transmission Patterns of Seasonal Influenza in China between 2010 and 2018
Source: Viruses. 2022 Sep 17;14(9):2063. doi: 10.3390/v14092063 (PMC9501233; doi:10.3390/v14092063)
Supplement: Supplementary file 1 [file viruses-14-02063-s001.zip › viruses-1882842-supplementary.pdf]

Supplementary Materials

Part 1. Methods

1.1 Human mobility proxies

Due to the lack of direct province-to-province transport data, estimations could only be used based on the available data. Firstly, the annual numbers of people traveling by railway, road, water, and air from 2010 to 2017 were obtained from *China Statistical Yearbook 2018* (available at: http://www.stats.gov.cn/tjsj/ndsj/2018/indexch.htm). Due to the lack of weekly transport data, the weekly human mobility proxies were used from *China Statistical Yearbook*, *China Transport Statistical Yearbook* and the China Railways Ticket website (12306.cn).

We took the number of passengers who traveled in 2016 as an example to explore the main inter-province transportation in China. The numbers of passengers traveling by railway, road, water, and air in China were 2.8×10^9, 1.5×10^{10}, 2.7×10^8, and 4.9×10^8, respectively. The number of passengers traveling by water and air was much less than the number of people traveling by road and railway, as only 3.5% of the road transport data were for inter-province transport (*China Transport Statistical Yearbook 2016*), which meant that there were 5.2×10^8 inter-province passengers traveling by road, much less than those traveling by railway. Given that in China, the main inter-province transport is the railway, we used the inter-province railway transport data as proxies for inter-province human mobility. The number of trains between provinces i and j was denoted by Tr_{ij} and were downloaded from the official China Railways Ticket website 12306.cn. Assumed that the number of people traveling on each train is the same, the daily number of people

who traveled from province i to province j is $T_{ij} = N_i Tr_{ij} / \sum_{i=1}^{31} Tr_{ij}$, where N_i is the mean daily number of people traveling by railway in province i.

1.2 Seasonality of Different Strains in Southern and Northern China through Wavelet Analysis

The wavelet transform of a time series x(t) can be considered the convolution of the series with a set of "wavelet daughters" generated by the mother wavelet by translation in the widths at different time positions (time-dependent spectral analysis of epidemiological time-series with wavelets). Generally, it is defined as:

$$W_x(a,\tau) = \frac{1}{\sqrt{a}} \int_{-\infty}^{\infty} x(t) \Psi^* (\frac{t-\tau}{a})\, dt.$$

where Ψ^*denotes the complex conjugate. Parameters a and τ are the scale factor and time shift, respectively. The set of scales determines the wavelet coverage of the original series in the frequency domain. The factor $1/\sqrt{a}$ normalizes the wavelets. The wavelet power spectrum is defined as the time–frequency (or time–period) wavelet energy density, which can be realized using the square of the local amplitude of any periodic component of the time series. In the present study, the wavelet power spectrum for each influenza strain was calculated, and the periodic features in northern and southern China were assessed.

1.3 Smoothing of the surveillance data

The smoothed surveillance data is obtained by the following equation:

$flu_s(1) = flu(1);$

$flu_s(i) = (flu(i-1) + flu(i) + flu(i+1))/3, 2 \leq i \leq i_{max} - 1;$

$flu_s(i_{max})= flu(i_{max})$.

where i is the epidemiological week number, i_{max} is the maximum epidemiological week, and $flu(i)$ and $flu_s(i)$ are the incidence of influenza in epidemiological week i before and after the smoothing, respectively.

1.4 Environmental space

A concept of environmental space was used to describe the suitability of a specific combination of the mean temperature and relative humidity for seasonal influenza transmission (Figure S3). To this end, the epidemic period from the onset to the end time as described above was identified first. Then, for each week within the epidemic period for each province, the number of specific combinations of the mean temperature and relative humidity was counted. Those numbers were then normalized by the total numbers measured as the suitable level. The higher the level for a specific combination of the mean temperature and relative humidity, the more suitable the combination for seasonal influenza transmission. The distribution density of suitable conditions for each region was also calculated by normalizing the total numbers from that specific region.

Part 2. Table S1-S5

Table S1. Transmission analysis within the same winter season for influenza A(H1N1)pdm09 based on sequences.

Route	Proportion±95% confident interval (greater probability) *
North to South	0.25±0.05(0.00)
South to North	0.69±0.13(1.00)

* Based on 100 bootstrap repeats.

Table S2. Transmission analysis within the same winter season for influenza B/Yamagata based on sequences.

Route	Proportion±95% confident interval (greater probability)
North to South	0.16±0.03(0.00)
South to North	0.58±0.09(1.00)

Table S3. Transmission analysis within the same winter season for influenza B/Victoria based on sequences.

Route	Proportion±95% confident interval (greater probability)
North to South	0.15±0.04(0.00)
South to North	0.57±0.08(1.00)

Table S4. Transmission analysis between seasons for influenza A(H3N2) based on sequences.

From	To<br>Proportion±95% confident interval (greater probability)	
	**South Winter**	**North Winter**
South Summer	0.52±0.05 (1.00)	0.50±0.05 (0.99)
North Summer	0.19±0.04 (0.00)	0.29±0.06 (0.01)
South Winter	0.16±0.04 (0.00)	0.10±0.04 (0.00)
North Winter	0.13±0.04 (0.00)	0.11±0.04 (0.00)

Table S5. Transmission analysis within the same season for influenza A(H3N2) based on sequences.

Route	Proportion±95% confident interval (greater probability)	
	**Winter**	**Summer**
North to South	0.21±0.04 (0.15)	0.11±0.03 (0.00)
South to North	0.29±0.06 (0.85)	0.46±0.07 (1.00)

Part 3. Figure S1-S3

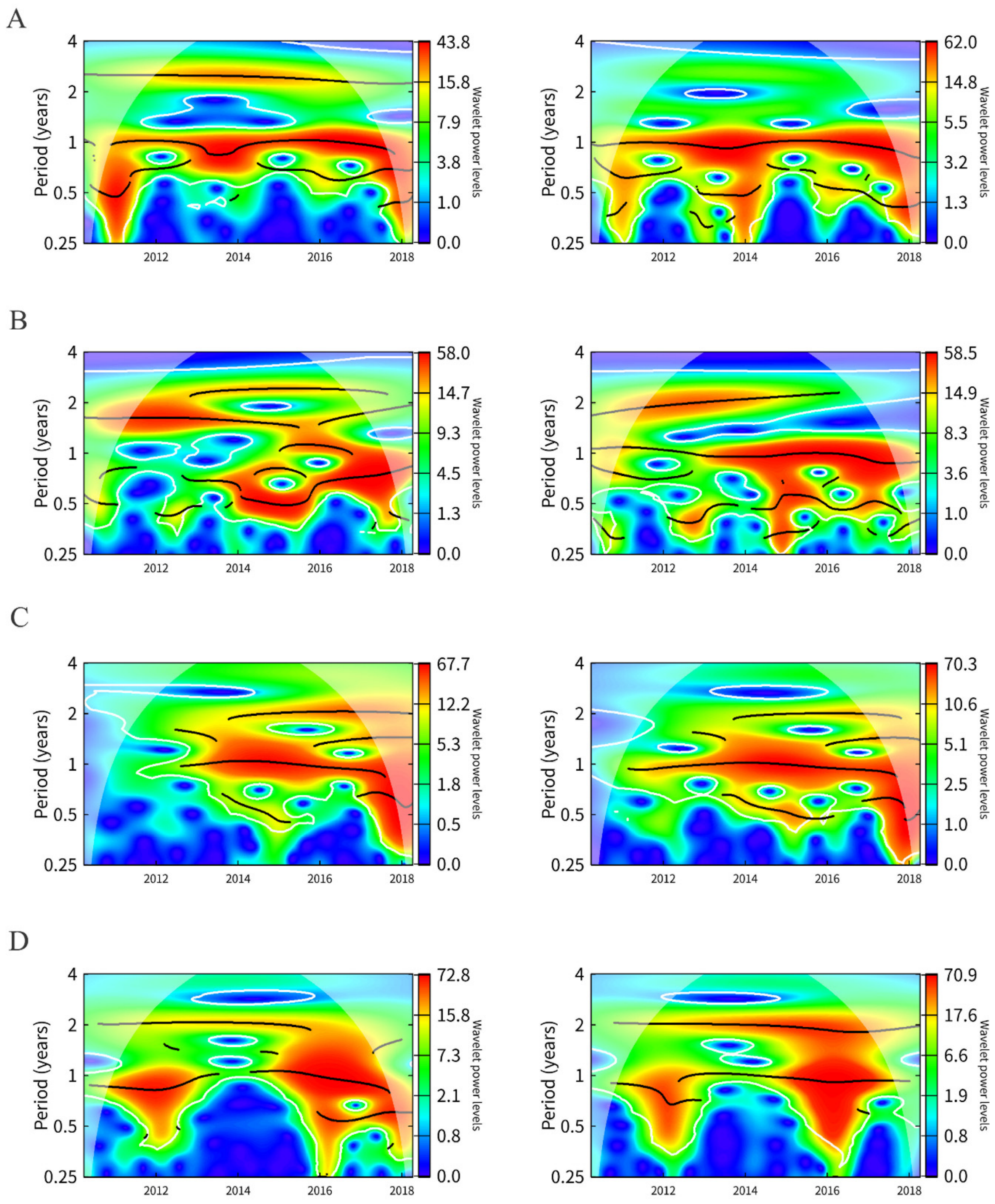

Figure S1. Wavelet analysis of the seasonality of influenza (A) A(H1N1)pdm09, (B) A(H3N2), (C) B/Yamagata, and (D)B/Victoria in southern (left) and northern (right) China, respectively.

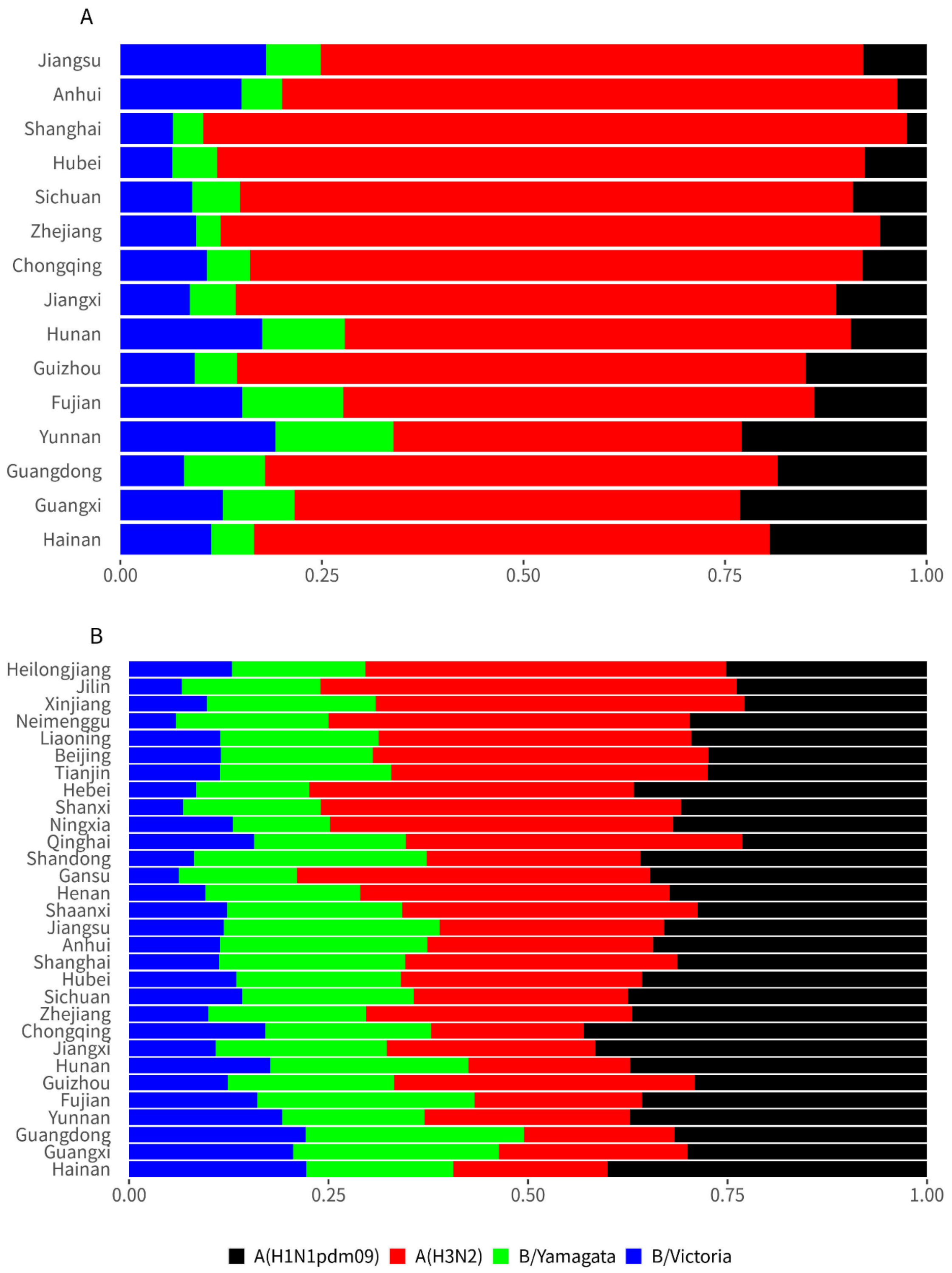

Figure S2. Prevalence of influenza strains in China: (A) summer season in southern China and (B) winter season across China. Provinces were sorted by increasing latitude from bottom to top.

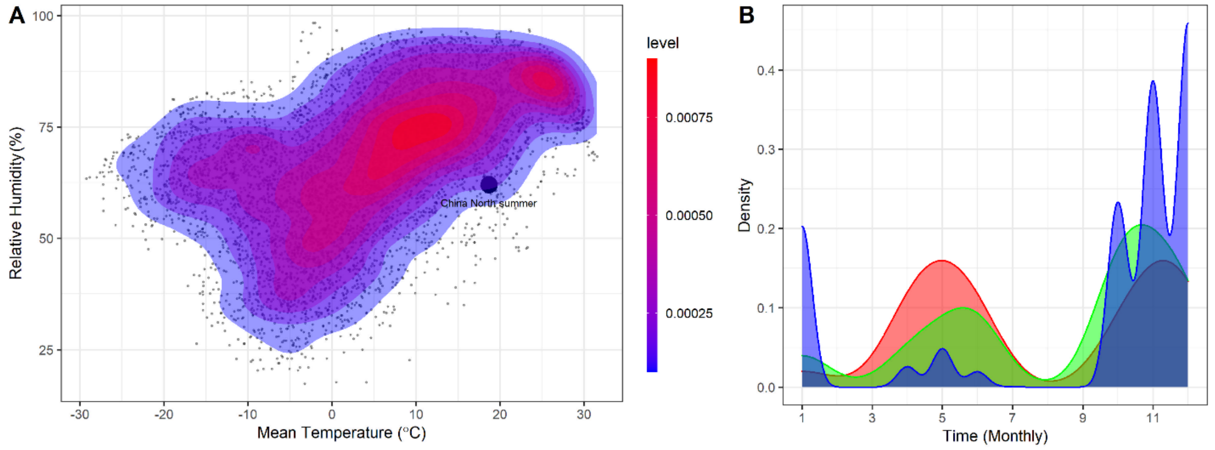

Figure S3. Environmental space for seasonal influenza A(H3N2) in China: (A) environment space levels illustrated based on mean temperature and relative humidity. The mean values in the summer season for provinces in northern China were marked as a black dot; (B) density curve for the three regions distinguished by different colors in Figure 3A.

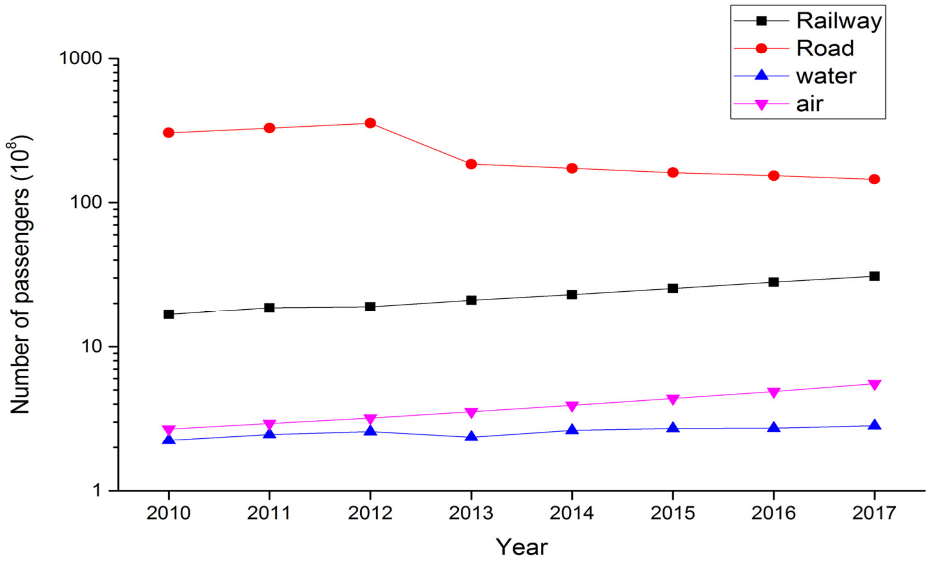

Figure S4. Number of passengers traveled by railway, road, water, and air from 2010 to 2017.
